# Exploring malaria vector diversity on the Amazon Frontier

**DOI:** 10.1186/s12936-018-2483-2

**Published:** 2018-09-27

**Authors:** Brian P. Bourke, Jan E. Conn, Tatiane M. P. de Oliveira, Leonardo S. M. Chaves, Eduardo S. Bergo, Gabriel Z. Laporta, Maria A. M. Sallum

**Affiliations:** 1 Department of Epidemiology, Faculty of Public Health, University of São Paulo, São Paulo, SP Brazil; 2 Wadsworth Center, New York State Department of Health, Slingerlands, NY 12159 USA; 3 Department of Biomedical Sciences, School of Public Health, State University of New York-Albany, Albany, NY 12222 USA; 4 Superintendência de Controle de Endemias, Secretaria de Estado da Saúde de São Paulo, Araraquara, SP Brazil; 5 Setor de Pós-graduação, Pesquisa e Inovação, Faculdade de Medicina do ABC, Santo André, SP Brazil

**Keywords:** Malaria, Species discovery, Mosquito, Anophelinae, Amazon, Deforestation

## Abstract

**Background:**

Deforestation in the Amazon and the social vulnerability of its settler communities has been associated with increased malaria incidence. The feeding biology of the most important malaria vectors in the region, notably *Nyssorhynchus darlingi*, compounds efforts to control vectors and reduce transmission of what has become known as “Frontier Malaria”. Exploring Anophelinae mosquito diversity is fundamental to understanding the species responsible for transmission and developing appropriate management and intervention strategies for malaria control in the Amazon River basin.

**Methods:**

This study describes Anophelinae mosquito diversity from settler communities affected by Frontier Malaria in the states of Acre, Amazonas and Rondônia by analysing *COI* gene data using cluster and tree-based species delimitation approaches.

**Results:**

In total, 270 specimens from collection sites were sequenced and these were combined with 151 reference (GenBank) sequences in the analysis to assist in species identification. Conservative estimates found that the number of species collected at these sites was between 23 (mPTP partition) and 27 (strict ABGD partition) species, up to 13 of which appeared to be new. *Nyssorhynchus triannulatus* and *Nyssorhynchus braziliensis* displayed exceptional levels of intraspecific genetic diversity but there was little to no support for putative species complex status.

**Conclusions:**

This study demonstrates that Anophelinae mosquito diversity continues to be underestimated in poorly sampled areas where frontier malaria is a major public health concern. The findings will help shape future studies of vector incrimination and transmission dynamics in these areas and support efforts to develop more effective vector control and transmission reduction strategies in settler communities in the Amazon River basin.

**Electronic supplementary material:**

The online version of this article (10.1186/s12936-018-2483-2) contains supplementary material, which is available to authorized users.

## Background

Deforestation is the permanent destruction of forests in order to harvest timber, develop farms and pasture, and build roads and urban areas. Many of the world’s most biologically diverse regions are now subject to the highest rates of deforestation and one of the most severely affected of these is the Amazon tropical rainforest. In Brazil over the past 50 years, deforestation in the Amazon has reached unprecedented levels, with estimated losses of between 4 and 29 thousand km^2^ annually [1]. During the same period, the human population in the region has increased from approximately 4 to 24 million [2]. Additional effects of regional deforestation include a dramatic loss of endemic species [3], creation of social conflict, contribution to global climate change, and elevated human vulnerability to socio-environmental conditions [4]. It is also having a negative impact on public health [5–7], and increases vector-borne diseases in the Amazon River basin [8]. One of the most important public health impacts is the increased transmission of *Plasmodium* species responsible for human malaria [9–13].

The increased incidence of *Plasmodium* infection in humans in Amazonian villages resulting from deforestation has been described as “Frontier Malaria”. This has been characterized as a temporal process comprised of epidemic, transition, and endemic phases [14, 15]. The epidemic phase involves changes in the natural forest landscape, including the biotic and abiotic conditions of forest larval habitats, that can favour species that are actively involved in the transmission of the *Plasmodium* to humans. Ongoing anthropogenic changes in the environment, which alter the abiotic characteristics and ecology of larval habitats [16], lead to an increase in abundance of the most important vector species and, ultimately, higher biting rates and *Plasmodium* infection in settler communities with poor housing conditions, lack of access to health services and low or no immunity to the pathogens [17]. The transition phase occurs several years after the initial settlements and sees gradual declines of transmission as a result of improved housing, infrastructure and public health services. The final endemic phase is reached when transmission reaches low and stable levels, normally within 10 years of the initial settlement. A developed public health service can achieve effective diagnosis and treatment of *Plasmodium falciparum* infection, due to symptoms occurring before gametocyte production. *Plasmodium vivax* infection, however, is more difficult to diagnose and treat, as the pathogen can exist at much lower densities [18] and persist as hypnozoites in the liver [19]. The only licensed treatments of the liver stage of *P. vivax* are primaquine and tafenoquine, but they are of limited use in some populations due to the adverse effect of acute haemolysis in people with G6PD deficiency [20, 21]. *Plasmodium falciparum* transmission emerges in the early stages of settlement and is joined by *P. vivax* in later stages, with the latter generally becoming more prevalent. An emerging public health consensus is that the dynamics of malaria in the Amazon River basin is unstable, with waves of disease emergence accompanied by explosive epidemics in many localities. These are usually associated with changes to natural environments and ecologies, waves of economic development, and migratory influxes between endemic and non-endemic areas. This dynamic process has challenged control programmes developed to mitigate the burden of malaria in the Amazon [22].

There are several vectors of malaria in the Neotropics that vary in importance at the local, regional and continental scale [23]. Although the highest rates of experimental *Plasmodium* infection have been found in *Nyssorhynchus aquasalis* and *Nyssorhynchus albitarsis* [24], with the latter presenting higher biting indices [25], *Nyssorhynchus darlingi* has the highest rates of natural infection and is seen as the most important malaria vector through much of South America [25, 26] (herein *Nyssorhynchus* is elevated from subgenus to genus rank, as proposed by Foster et al. [27]). This species is the most anthropophilic, endophilic and opportunistic vector, and is relatively abundant in the more populated localities of the Amazon [16]. It is also strongly associated with human environments in deforested areas in the Amazon [28, 29].

A great variety of potential vectors have been recorded in the deforested areas of the Amazon [30–32], but the frequent occurrence of morphologically indistinguishable sibling species, which form species complexes [33], means that vectors are often misidentified or cryptic species boundaries go undetected [34–36]. Some vectors, such as *Nyssorhynchus triannulatus* and *Anopheles peryassui*, inhabit the forest edge, away from domestic environments and conventional vector control activities, but may be associated with human activity such as deforestation [37]. Failure to properly elucidate the diversity present in vector species complexes at locations where forest gives way to frontier settlements will likely obstruct the understanding of vector ecology and the *Plasmodium* transmission cycle associated with deforestation in the Amazon and impede efforts to develop strategies to control frontier malaria [10, 38]. It is, therefore, essential to develop and employ a range of tools to discover potential vectors in malaria endemic areas.

Molecular tools are commonly employed to identify known species and delimit new species [39]. DNA barcoding is now seen as an important first step in biodiversity assessment and sorting specimens into tentative species, and *COI* the marker of choice because of the availability of universal primers that amplify across a diverse range of species, and the relatively low intraspecific and high interspecific divergence that occurs at this locus. It is clear that barcoding approaches are helping to resolve morphologically indistinguishable species within Anophelinae [31, 34, 40, 41].

Although barcoding studies frequently employ phylogenetic (and normally NJ) trees to delimit species, resolving intraspecific from interspecific phylogenetic structure is frequently subject to observer bias [42, 43]. Approaches explicitly designed for empirically and objectively delimiting species boundaries have been developed, among which are clustering and tree-based approaches, which do not require a priori definitions of taxa [44] or even a threshold for intraspecific diversity [45]. The use of such approaches can therefore allow species richness and delimitation to be independently estimated and tested against a priori species morphological definition.

The current study aims to describe Anophelinae species diversity in rural settlements affected by frontier malaria in the Amazon River basin, employing clustering-based (Automatic Barcode Gap Discovery; [44]) and tree-based (multi-rate Poisson tree processes; [45]) species delimitation approaches. The ability to describe diversity and delimit species in this setting is fundamental to understanding the vectors driving malaria transmission and allows for the development of more effective vector control and management strategies in frontier settlements in the Amazon River basin. In the study we pay particular attention to vector complexes and the potential for these to harbour new morphologically similar and potentially medically important species.

## Methods

### Mosquito collection

**Fig. 1:**
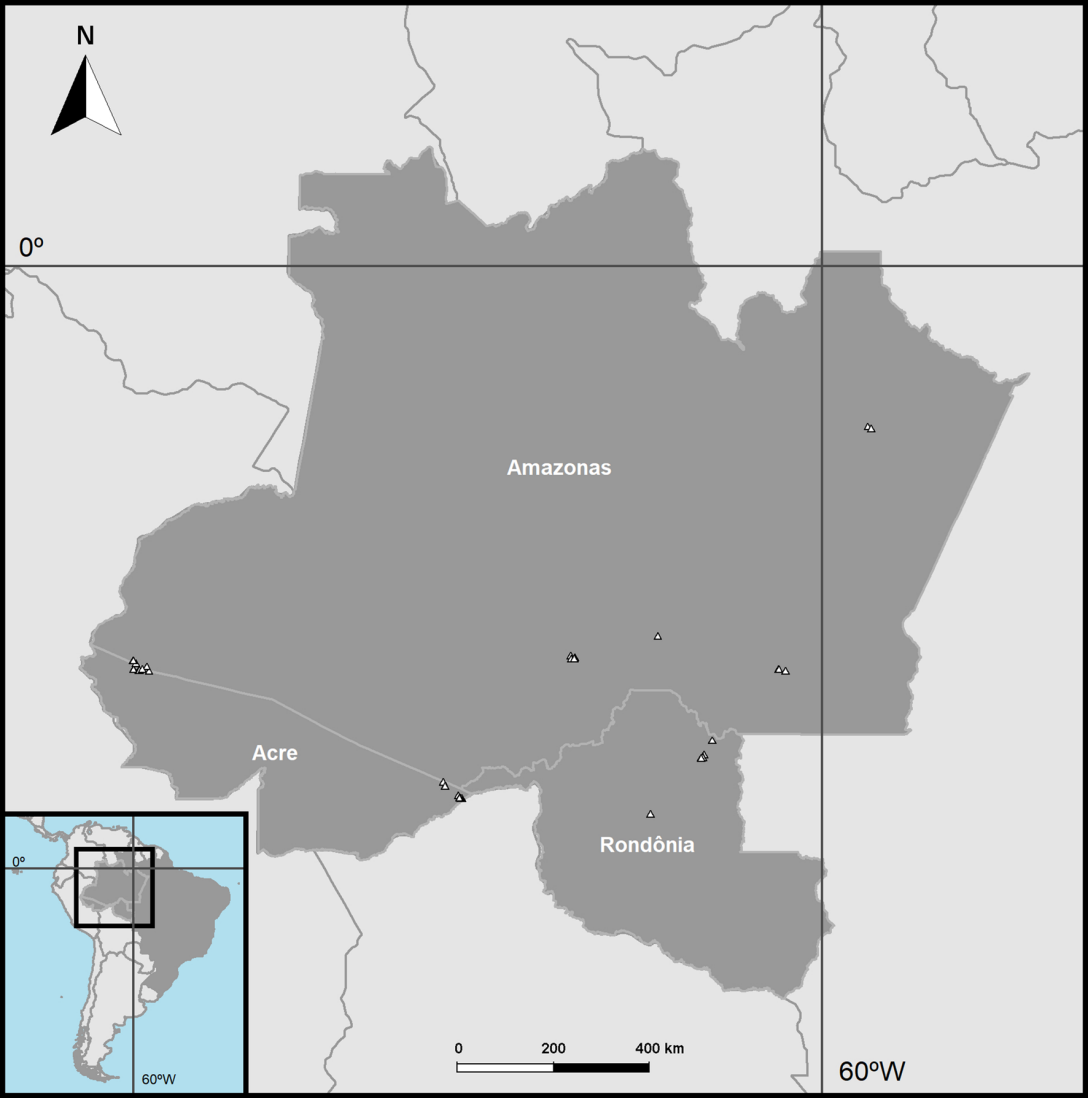
Specimen collection sites in the states of Acre, Amazonas and Rondônia. Collection sites are represented by triangles

In total, 270 mosquito specimens from 47 collection sites in the Brazilian states of Acre, Amazonas and Rondônia were included in this study (Fig. 1). Collection sites were selected based on a high annual malaria parasite index (annual parasite index, API ≥ 50) in 2015 or 2016 and levels of forest cover depletion greater than 10%, assuming this was representative of a human colonization process in the Amazon Forest. Field females were collected at the forest edge by either Shannon trap or human landing interception, using a small, manual aspirator. Females were euthanized with ethyl acetate vapors inside a plastic chamber in the field, and kept in silica gel and separated hourly. Collections at each site were made on a single day from 06:00 p.m. to 06:00 a.m., during the months of January, April, May, August, and October 2015. Specimens were identified from the Genus *Anopheles* and the recently elevated Genus *Nyssorhynchus* [27]. After species identification, females were kept individually frozen at − 80 °C. One or two legs were removed with small, pointed scissors and used for sequencing the 658 base pair *COI* barcode region of the mitochondrial genome. One hundred and fifty-nine sequences from thirty species were downloaded from GenBank and used in the phylogenetic analysis as references (Additional files 1, 2).

### DNA extraction

Genomic DNA extraction was performed from 1 or 2 legs of each Anophelinae specimen (Additional file 1). Each extraction was carried out as follows: legs were macerated in 10 µl of NaCl 0.9% with an autoclaved pistil; 20 µl of Chelex-100 5% was added then, vortexed; the solution was incubated at 99 °C for 10 min and then centrifuged at 13,000 rpm for 5 min at 25 °C; the supernatant was recovered and an aliquot was frozen at − 20 °C and the remaining DNA at − 70 °C in the entomological frozen collection of the Faculdade de Saúde Pública, São Paulo, Brazil.

### DNA amplification

Primers described by Folmer et al. [46] were used to amplify the barcode region of the cytochrome c oxidase gene (*COI*). Each reaction was performed with final volume of 25 µl containing 2 µl of DNA extracted by Chelex, 1× PCR Buffer (Invitrogen), 1.5 mM MgCl_2_ (Invitrogen), 0.2 mM each dNTPs (Amresco), 0.1 μM each primer (LCO1490 5′-GGTCAACAAATCATAAAGATATTGG-3′ and HCO2198 5′-TAAACTTCAGGGTGACCAAAAAATCA-3′), 0.625 U Taq Platinum polymerase (Invitrogen) and the remaining volume of ultra-pure water. The thermocycler conditions were 94 °C for 3 min, 5 cycles of 94 °C for 30 s, 45 °C for 90 s, 68 °C for 60 s, followed by 35 cycles of 94 °C for 30 s, 51 °C for 30 s, 68 °C for 60 s and a final extension at 68 °C for 10 min [47]. PCR products were purified using PEG precipitation (20% polyethyleneglycol 8000/2.5 M NaCl).

### Sequencing and alignment

Sequencing reactions proceeded in both directions using a Big Dye Terminator cycle sequencing kit v3.1 (Applied Biosystems, Foster City, CA, USA) and Applied Biosystems 3130 DNA Analyzer (Applied Biosystems). Sequencing reactions were carried out with the same set of PCR primers. The sequencing products were purified using Sephadex G50 columns (GE Healthcare) and analysed in an Applied Biosystems 3130 DNA Analyser (PE Applied Biosystems). Sequences were edited in Sequencher v.4.9 software (Genes Codes Corporation, Ann Arbor, MI, USA), and the primer regions removed. In addition to these novel sequences, 162 sequences from GenBank were also included, to serve as references in the phylogenetic and species delimitation analysis. The *COI* gene sequences were aligned first by nucleotides using the Muscle algorithm [48] implemented in SeaView [49], and then by amino acid using TranslatorX [50].

### Phylogenetic analysis

A Bayesian phylogenetic analysis was applied to sequences using a codon partitioning scheme to allow different partitions to have their own model characteristics (composition, rate matrix and among-site variation) and to allow for among-partition rate variation. Optimal evolutionary models for the sequences were determined for each partition using the corrected Akaike Information Criterion (AICc) in jModelTest 2 [51]. With the exception of 2 singletons, codon position 2 was invariable and so was excluded from phylogenetic analyses. The optimal models for codon positions 1 and 3 were GTR + G and GTR + G + I, respectively. All Bayesian phylogenetic analyses were performed using MrBayes 3.2 [52] at the CIPRES Science Gateway [53] and each analysis consisted of two simultaneous runs, which were then repeated to provide confirmation of convergence of posterior probability distribution. Each Bayesian phylogenetic analysis was run for fifty million generations, with the first twenty-five million generations being discarded as burn-in. The Metropolis-coupled Markov chain Monte Carlo strategy was used with four heated chains; adequate mixing was achieved by setting the chain temperature to 0.03. Convergence of topology between the two simultaneous runs was monitored using the average standard deviation of split frequencies—this index consistently fell to below 0.01 in the post-burn-in samples. Convergence was also monitored by noting that the potential scale reduction factor values were all approximately 1.0 in the post-burn-in samples. Consensus trees were constructed containing nodes with posterior probability support of at least 70% using SumTrees [54, 55]. Pairwise Kimura-two-parameter (K2P) [56] distances were calculated from the *COI* alignment using APE [57].

### Species delimitation

The first species delimitation method employed was the Automatic Barcode Gap Discovery—ABGD [44], which is a distance-based method that assigns sequences into potential species based on the detection of a barcoding gap (interspecific variation > intraspecific variation). It does not require a priori species designation but does require the setting of an intraspecific threshold (set to the range P_min_ = 0.005–P_max_ 0.1), below which interspecific diversity will not be detected.

The second species delimitation method employed was the multi-rate Poisson tree process—mPTP (multi-rate Poisson tree processes; [45]), which is a “phylogeny-aware” method that uses differences in mutation rate in a phylogenetic tree to resolve interspecific from intraspecific diversity. Unlike the previous method, it does not rely on a priori distance thresholds but does require a bifurcating non-ultrametric phylogenetic tree for input. A maximum likelihood (ML) tree was generated using RAxML-HPC BlackBox 8.2.10 [58] on the CIPRES Science Gateway [53] using gene partitioning by codon (with the 2nd codon position excluded due to invariability) under the model GTR + G, based on 1000 bootstrap replicates.

## Results

### Phylogenetics

A total of 421 sequences (270 newly sequenced from Amazonian rural settlement collections, and 151 downloaded from GenBank databases) from the genus *Anopheles* (in the Arribalzagia series from subgenus *Anopheles*) and genus *Nyssorhynchus* (in the Albimanus and Argyritarsis Series) were included in the analysis (Additional file 1). After alignment these yielded 359 *COI* haplotypes of 658 base pairs in length. *Chagasia bonneae* (GenBank accession no. KF671010) was also included to serve as an outgroup taxon.

**Fig. 2:**
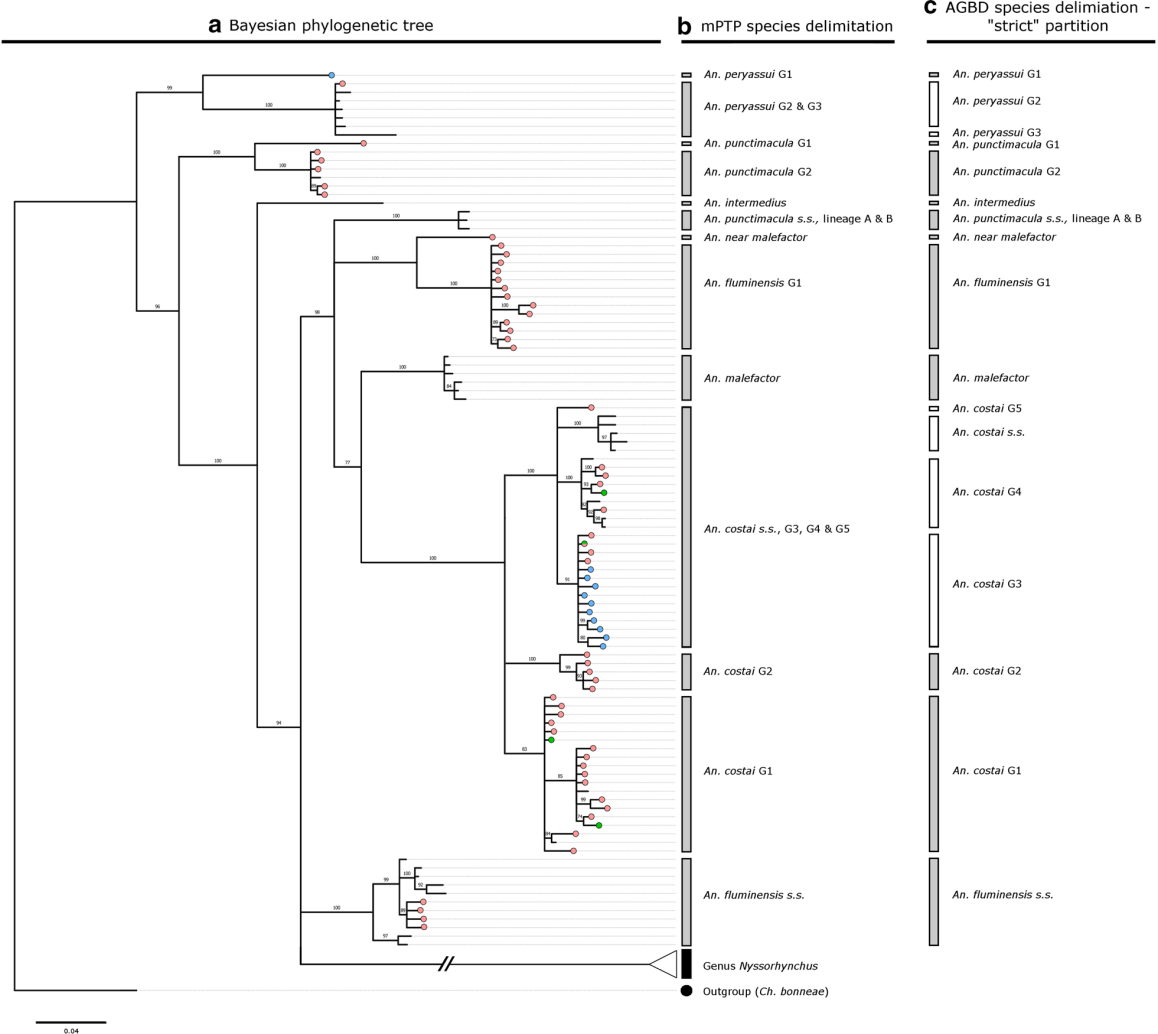
Phylogenetic and species delimitation analysis detailing collection specimens and reference sequences belonging to the Genus *Anopheles*. **a** Consensus tree from a Bayesian phylogenetic analysis using the *COI* gene. Numbers at branches indicate Bayesian Posterior Probability (≥ 70%). Haplotypes from collection specimens are coloured: Red = Acre, Green = Amazonas, Blue = Rondônia. *Chagasia bonneae* was included as an outgroup taxon, **b** Multi-rate Poisson Tree Process (mPTP) species delimitation analysis, **c** Automatic Barcode Gap Discovery (ABGD) analysis

**Fig. 3:**
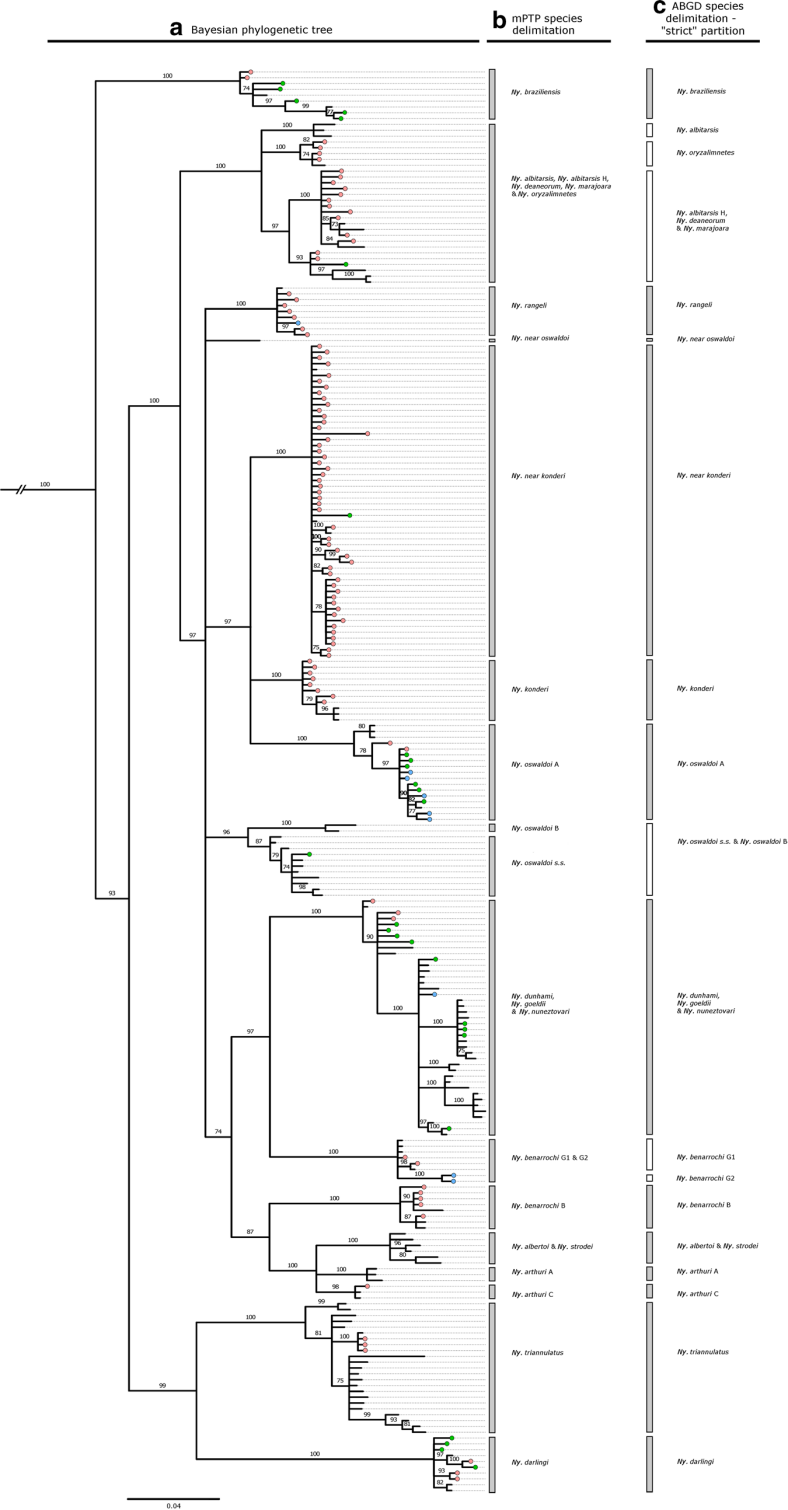
Phylogenetic and species delimitation analysis detailing collection specimens and reference sequences belonging to the Genus *Nyssorhynchus*. **a** Consensus tree from a Bayesian phylogenetic analysis using the *COI* gene. Numbers at branches indicate Bayesian posterior probability (≥ 70%). Haplotypes from collection specimens are coloured: Red = Acre, Green = Amazonas, Blue = Rondônia. *Chagasia bonneae* was included as an outgroup taxon, **b** Multi-rate Poisson Tree Process (mPTP) species delimitation analysis, **c** Automatic Barcode Gap Discovery (ABGD) analysis

Results of the Bayesian analysis found no evidence for reciprocal monophyly between the subgenera *Anopheles* and *Nyssorhynchus* (Figs. 2a and 3a). The subgenus *Anopheles* is paraphyletic with respect to *Nyssorhynchus*, with two of its clades forming a three-way polytomy with the latter (BPP 85%; Fig. 2a).

#### Genus *Anopheles* (see Fig. 2a)

*Anopheles peryassui* was recovered as a strongly supported but highly diverse monophyletic clade (99% BPP). It contained a haplotype (Sample id: RO38_2; Accession number: MF381690) that is at least 7.4% different (K2P distance) from its conspecifics. Although *Anopheles intermedius* from the state of São Paulo was included in the analysis, it appeared from the phylogenetic tree (and was confirmed in subsequent species delimitation analysis) that this sequence was the sole representative of *An. intermedius* in this study.

*Anopheles punctimacula* formed two strongly supported monophyletic clades (both with BPP of 100%) that appear to have very different evolutionary histories. The first clade was sister (96% BPP) to a clade containing all but one (*An*. *peryassui*) of the ingroup species. This first clade is also highly diverse, containing a haplotype (*Anopheles punctimacula* AC22_64) that is at least 5.9% different (K2P distance) from other haplotypes within the clade. The second *An. punctimacula* clade contains haplotypes from *An. punctimacula* sensu stricto/lineage A and *An. punctimacula* lineage B detailed in Loaiza et al. [36]. It is recovered in a disparate part of tree from the first *An. punctimacula* clade.

*Anopheles fluminensis* was recovered as two phylogenetically distant clades (both with BPP of 100%). The first clade includes *An. fluminensis* from the type locality in Rio de Janeiro, and herein considered sensu stricto. It is found on a three-way polytomy with the *Nyssorhynchus* clade and a clade containing *Anopheles costai*, *Anopheles malefactor*, *An*. *punctimacula* s.s./lineage A, *An. punctimacula* lineage B and the second *An. fluminensis* clade. This second clade was found to be sister (100% BPP) to a highly distinct lineage of *An*. *malefactor*. There is a difference of 8.0–9.3% (K2P distance) between these two *An. fluminensis* clades. The highly distinct *An*. *malefactor* lineage is herein denoted *An*. near *malefactor* and is clearly resolved from *An. malefactor* sensu lato by a difference of 8.4–9.0% (K2P distance).

*Anopheles costai* was resolved (100% BPP) as a highly diverse clade (0.0–7.8% K2P distance). The *An. costai* haplotype relationships within this clade are suggestive of between three and six lineages, but the lineage composition of this clade will be more clearly described in the results from species delimitation below.

#### Genus *Nyssorhynchus* (see Fig. 3a)

Members of the Oswaldoi Subgroup included in this study are the Oswaldoi Complex, the Konderi Complex and the Nuneztovari Complex. Members of the Oswaldoi Complex in this study are *Nyssorhynchus oswaldoi* sensu stricto, *Ny. oswaldoi* A, *Ny. oswaldoi* B, and *Nyssorhynchus rangeli*. One of these, *Ny. oswaldoi* A (100% BPP), forms a monophyletic clade within the three-way polytomy (97% BPP) with *Nyssorhynchus konderi* and *Ny.* near *konderi*. *Nyssorhynchus rangeli* (100% BPP) is a monophyletic clade on a five-way polytomy (97% BPP) that also contains a clade (96% BPP) comprised of *Ny. oswaldoi* s.s. (87% BPP) and *Ny. oswaldoi* B (100% BPP), which are reciprocally monophyletic. The two members of the Konderi Complex, *Ny. konderi* (100% BPP) and *Ny*. near *konderi* (100% BPP), each form monophyletic clades within the three-way polytomy described above. The Nuneztovari Complex (100% BPP) is composed of *Nyssorhynchus dunhami* and several lineages from *Nyssorhynchus goeldii* and *Nyssorhynchus nuneztovari*. Of the five *Ny. goeldii* and *Ny. nuneztovari* clades (Groups I–V) detailed in Scarpassa et al. [40], *Ny. goeldii* Group I (100% BPP), *Ny. nuneztovari* Group II (100% BPP) and Group IV (100% BPP) are found to be monophyletic and *Ny. goeldii* Group III are *Ny. goeldii* Group V are paraphyletic. *Nyssorhynchus dunhami* is also paraphyletic with respect to other members of the Nuneztovari Complex.

Sequences from six members of the Strodei Subgroup are included in this study. *Nyssorhynchus benarrochi* is resolved into two quite distinct clades. The first is identified from Acre and Rondônia (100% BPP) and is found as a sister (97% BPP) to the *Ny. nuneztovari* complex. The second is a distantly related *Ny. benarrochi* B clade (100% BPP) found to be sister (87% BPP) to the *Nyssorhynchus strodei* subgroup (100% BPP). *Nyssorhynchus arthuri* A (100% BPP) and *Ny. arthuri* C (98% BPP) formed distinct clades on a three-way polytomy (100% BPP) with the *Ny. strodei*/*Nyssorhynchus albertoi* clade (100% BPP), which are unresolved.

The Albitarsis Series is comprised of the Albitarsis Group, made up of the Albitarsis Complex, and the Braziliensis Group, made up of *Nyssorhynchus braziliensis*. In this study, the Albitarsis Complex is resolved as a monophyletic clade (100% BPP), and all (*Ny. albitarsis*, 100% BPP; *Nyssorhynchus deaneorum*, 100% BPP; *Nyssorhynchus marajoara*, 97% BPP; *Nyssorhynchus oryzalimnetes*, 100% BPP) but one (*Ny. albitarsis* H) of its multiple species form monophyletic clades. *Nyssorhynchus braziliensis* (100% BPP) is resolved from all other species in *Nyssorhynchus* (100% BPP) and shows high levels of haplotype diversity, up to 3.3% (K2P distance).

*Nyssorhynchus darlingi* (100% BPP) is resolved as a sister species (99% BPP) to the highly diverse *Ny. triannulatus* clade (100% BPP), with intraspecific diversity of up to 3.8% (K2P distance).

### Species identification

**Fig. 4:**
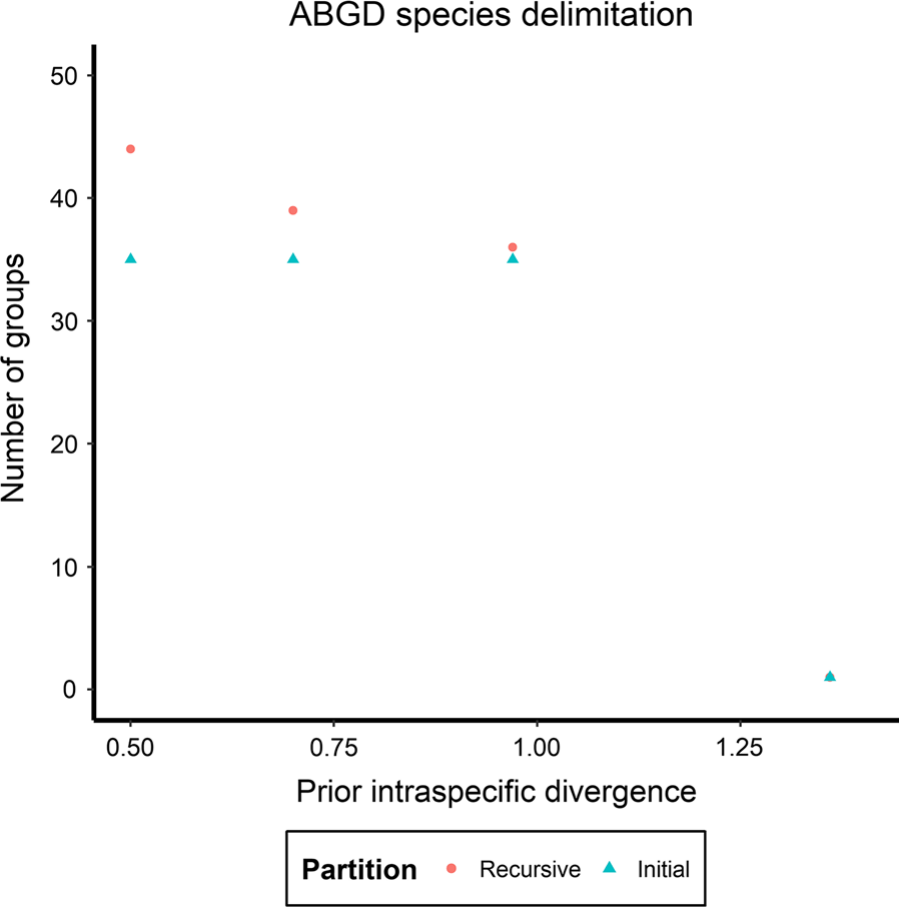
Partitions obtained from Automatic Barcode Gap Discovery (ABGD) analysis of collection specimens and reference sequences

Collection and reference specimens are delimited into 30 “species” groups by the mPTP analysis (Additional file 3; Figs. 2b, 3b). The ABGD analysis provided three possible partitions for these specimens before grouping them together as a whole (Fig. 4). The most conservative partitioning produces 36 groups (Additional file 4; Figs. 2c, 3c) and is herein referred to as the “strict” partition. The most fragmented partitioning produces 44 groups (Additional file 5) and is herein referred to as the “relaxed” partition.

#### Genus *Anopheles* (see Fig. 2b, c)

Under the mPTP (phylogeny-aware) analysis, 3 groups are delineated for *An. costai*. The first group, herein denoted *An. costai* G1, has a large distribution, and is identified in the states of Acre and Amazonas and the Colombian departments of Putumayo (Accession number: JX205128) and Amazonas (Accession number: KF698865). This group has good support in the Bayesian phylogenetic tree (89% BPP). The second delineated group, herein denoted *An. costai* G2, is only identified from rural settlements in Acre and is well supported in the Bayesian phylogenetic analysis (100% BPP). Both *An. costai* G1 and *An. costai* G2 are consistently delineated in the strict and relaxed ABGD species partition. The third group includes *An. costai* sensu stricto haplotypes from the state of São Paulo (the type locality; Field Specimen ID: SP02_17_3) as well as haplotypes from other locations in Acre, Amazonas, Bahia, Rondônia, and the Colombian departments of Meta (Accession numbers: HM022403 and HM022404) and Putumayo (Accession number: JX205127). This group is further partitioned into four groups in both the strict and relaxed ABGD (K2P distance-based) species partitions. These groups are herein denoted *An. costai* sensu stricto (identified from Bahia and São Paulo), *An. costai* G3 (identified from Acre, Amazonas and Rondônia), *An. costai* G4 (identified from Acre, Amazonas and Colombia) and *An. costai* G5 (identified from Acre only).

*Anopheles fluminensis* is consistently partitioned into two groups across all species delimitation analyses, and these groups represent the two polyphyletic clades identified in the Bayesian phylogenetic analysis. The first group includes specimens from five rural settlements in Acre in addition to specimens from Rio de Janeiro (the type locality—Reserva Biológica do Tinguá; Accession number: MF381677), São Paulo and Ecuador, but denoted *An*. nr *fluminensis* in Linton et al. [59]. This group is tentatively denoted *An. fluminensis* sensu stricto, although there is considerable intra-group diversity that ranges from 0.6 to 3.3% (K2P distances), with the São Paulo and Rio de Janeiro haplotypes (0.6% different) having distances of between 2.0 and 3.3% with the other members of the group. The second group is comprised of specimens collected from eight rural settlements in Acre, and is herein denoted *An. fluminensis* G1. The two groups differ by between 7.7 and 9.3% (K2P distances). *Anopheles malefactor* is also partitioned into two groups across all species delimitation analyses and is again consistent with the polyphyly observed to the phylogenetic analysis. The first group is *An. malefactor*, of González et al. [41] and Gómez et al. [31], whereas the second is herein denoted *An*. near *malefactor*, and they differ by between 8.4 and 9.0% (K2P distance).

Both mPTP and ABGD species delimitation approaches found three groups in *An. punctimacula*. Two of these groups are found in the highly diverse *An. punctimacula* clade and herein are denoted *An. punctimacula* G1 (a singleton) from Acre and *An. punctimacula* G2 from Acre and the Colombian department of Putumayo (Accession number: JX205122). The third group is made up of the *An. punctimacula* ss, *An. punctimacula* lineage A and *An. punctimacula* lineage B (from [36]).

The mPTP species delimitation approach identified two groups in *An. peryassui*. The first group, herein denoted *An. peryassui* G1, is represented by a single haplotype from Rondônia (Sample id: RO38_2; Accession number: MF381690), and identified as highly divergent (7.4% K2P distance) in the phylogenetic analysis. The second group is made up of haplotypes from Acre, Para, the Colombian departments of Amazonas (Accession numbers: KF698875–KF698877; [31]) and Meta (HM022405), and the state of Amapa, with this latter haplotype delineated as a third group in both the strict and relaxed partitions of the ABGD species delimitation. The *An. intermedius* singleton from São Paulo was separately delimited across all species delimitation analyses.

#### Genus *Nyssorhynchus* (see Fig. 3b, c)

Within the Strodei Subgroup, *Ny. albertoi* and *Ny. strodei* are not delineated in either mPTP or ABGD analyses. *Ny. arthuri* A and *Ny. arthuri* C are delineated across all analyses but only *Ny. arthuri* C was collected in this study at a rural settlement in Rondônia. Two groups are identified from the Benarrochi Complex. The first is *Ny. benarrochi* B, which is delineated in the mPTP analysis and from the ABGD strict partition (but splits into 4 groups in ABGD relaxed partition). *Nyssorhynchus benarrochi* B was collected from five rural settlement in Acre. The second group is delimited in the mPTP analysis, but splits into two further groups, herein denoted *Ny. benarrochi* G1 and *Ny. benarrochi* G2, in both the strict and relaxed partition from the ABGD analyses. *Nyssorhynchus benarrochi* G1 was collected from a rural settlement in Acre, while *Ny. benarrochi* G2 was collected from three rural settlements in Rondônia.

The sole representative from the Argyritarsis Series included in this study is *Ny. darlingi*. This species is clearly delineated across all analyses, and identified from six rural settlements in Acre and Amazonas. With respect to the Albitarsis Series, *Ny. braziliensis* is delimited in mPTP analysis and in the ABGD strict partition. However, it decomposes into five separate groups (of which 3 were singletons) in the ABGD relaxed partition. Within the Albitarsis Complex, only *Ny. albitarsis* and *Ny. oryzalimnetes* are delimited in analyses (ABGD strict and relaxed partitions). *Nyssorhynchus albitarsis*, *Ny. deaneorum* and *Ny. marajoara* are consistently grouped together in all species delimitation analyses.

The Oswaldoi Subgroup contains the Konderi, Oswaldoi and Nuneztovari Complexes, all of which are represented in this study. Species from this subgroup that can be clearly delimited across all species delimitation analyses are *Ny. rangeli* (rural settlements in Acre and Rondônia), *Ny. oswaldoi* A (rural settlements in Acre, Amazonas, Rondônia), *Ny. konderi* and *Ny*. near *konderi*. In the case of *Ny*. *konderi*, the “*An. konderi* of Amapá” (GenBank accession: JF437967 and JF437968) in Motoki et al. [60], the “*An. konderi* of Sallum” (GenBank accession: KF809030–033) in Ruiz-Lopez et al. [61] and “*An. konderi*” (GenBank accession: JF923716) in Saraiva et al. [34] all belong to this lineage. This lineage was collected in rural settlements in Acre as well as from sites in Amapá. In the case of *Ny*. near *konderi*, the “*An*. *konderi* of Acre” (GenBank accession: JF437965) in Motoki et al. [60] and the “*An*. near *konderi*” (GenBank accession: KF670997 and KF809137) in Ruiz-Lopez et al. [61] belong to this lineage. This lineage was collected from rural settlements in Acre and Amazonas. *Nyssorhynchus oswaldo*i s.s. and *Ny. oswaldoi* B are delimited in the mPTP analysis and the ABGD relaxed partition, but not in the ABGD strict partition. This species delimitation (and the phylogenetic analysis) suggests the “*An. konderi* of Paraná and Rondônia” (GenBank accession: JF437969–JF437974) in Motoki et al. [60] belongs to *Ny. oswaldoi* s.s. (GenBank accession: KF809126 and KF809128). *Nyssorhynchus oswaldoi* s.s. was collected from a single rural settlement in Amazonas, whereas *Ny. oswaldoi* B was absent from all rural settlements in our study. None of the members of the *Ny*. *nuneztovari* complex are resolved in any of the species delimitation analyses. Finally, *Ny. triannulatus* is delineated as a single group across all analyses, and identified from a single rural settlement in Acre.

#### Summary of species delimitation for collection specimens

**Fig. 5:**
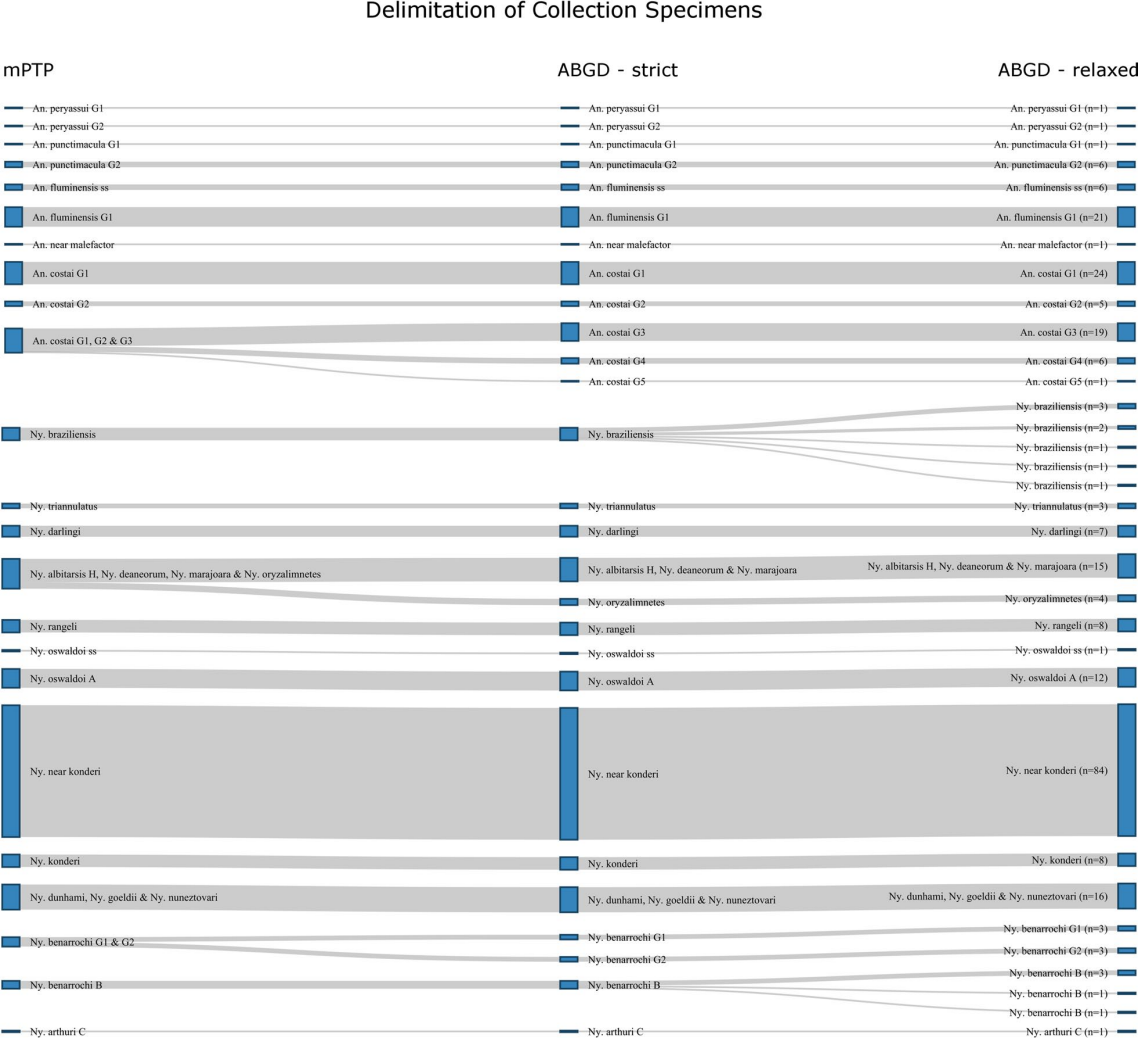
Summary of all species delimitation analysis of collection specimens from Acre, Amazonas and Rondônia

The most conservative species delimitation approach used in this study (mPTP) delimits at least 23 putative species from these collection sites (Fig. 5), and 30 from the study as a whole i.e. including reference sequences. The strict clustering-based (ABGD) partition delimits 27 putative species (Fig. 5) and 36 from the study as a whole, while the relaxed clustering-based (ABGD) partition delimits 33 putative species (Fig. 5) and 44 from the study as a whole.

## Discussion

The pattern of deforestation and anthropogenic changes in natural environments, especially in the Amazon tropical rainforest, and the insidious poverty of settlers living in precarious conditions, have been associated with increases in the incidence of malaria [62]. Compounding this problem in areas of the Amazon River basin with active malaria is the primary vector *Ny. darlingi*, which is a generalist and opportunistic species that can blood-feed both indoors and outdoors complicating the dynamics of transmission [63]. There are also other important vectors involved in malaria transmission in the region, depending on the local ecological conditions. Considering the urgent need for studies focusing on practical malaria that includes accurate identification of malaria vectors, this study demonstrates the capability of the barcode region of the *COI* mitochondrial gene and commonly used species delineation approaches to delimit a diverse range of Anophelinae species found in rural settlements affected by frontier malaria in the Amazon River basin. After assigning collection specimens to 19 morphospecies, conservative estimates delimit between 23 and 27 potential species, up to 13 of which appear to be new species.

Several species are well resolved in our phylogenetic tree and consistently delineated as coherent groups across analyses. These include *Ny. darlingi*, *Ny. rangeli* and *An. intermedius*. *Ny. darlingi* is the primary vector of malaria in the Amazon River basin [16]. It is a highly anthropophilic species and can vary from endophagy to exophagy, and endophily to exophily [64]. As such, vector control can be extremely challenging and the presence of this species at six rural settlements in Acre and Amazonas is significant for malaria transmission and vector control. In addition, *Ny. rangeli* has been found to be important in local malaria transmission in Colombia [65] and its larval habitat is associated with human-modified environments [66]. Its presence in rural settlements in Acre and Rondônia may indicate a potential role in frontier malaria at these localities. *Anopheles intermedius* sequence data (a singleton from São Paulo state) was included in the analysis as a reference as it is a potential malaria vector in the region, with natural infection detected in Amapa [67], Pará [68], and French Guiana [69]. However, this species was not detected at any of the study’s collection sites.

Although *Ny. braziliensis* was recovered as a monophyletic species and singularly grouped in both species delineation approaches, the species appeared to be highly diverse and partitioned into multiple groups in the relaxed ABGD partition. Insufficient sampling meant that we could not attribute any significance to this structure. The local abundance of this species in the Amazon River basin varies considerably [37, 67, 70] but it appears to be common in human-modified environments [71]. The species is highly anthropophilic [37] and it has been found naturally infected with *P*. *malariae* [70], *P*. *vivax* [25, 72] and *P*. *falciparum* [25, 73]. Its presence in rural settlements may therefore have implications for frontier malaria transmission.

To date, there is little genetic data available for *An*. *peryassui*, whose range covers most of northern South America. There is some evidence to suggest that the species may play a role in malaria transmission, with natural infection by *P. falciparum* and *P. vivax* reported in the state of Amazonas [74]. The current study included specimen reference data from Colombia in Gómez et al. [31], the only other source of *An*. *peryassui* genetic data available in GenBank. Results of the analyses demonstrated that *An*. *peryassui* appears to exist as a species complex with at least two (and possibly three) lineages identified, with the first found in Rondônia and the second found in Acre, Amapa, Colombia and Para (with the Amapa haplotype possibly splitting into a third lineage). However, the systematics of this complex remains poorly understood, because of the lack of material from the type locality in Rio Bonito in the state of Minas Gerais.

The genetic diversity of *An. fluminensis* has also been poorly explored. This lack of interest may be due to the understanding that *An. fluminensis* is not an important malaria vector [75], although specimens identified as *An*. near *fluminensis* have been incriminated as a malaria vector in the department of Junin, Peru [76] and more recently the species was found naturally infected by *Plasmodium malariae* in the Atlantic Forest of São Paulo, Brazil [77]. A study of mosquito diversity in the Ecuadorian Amazon found some genetic support for *An*. near *fluminensis*, demonstrating a 2.75–3.77% difference between sequences from the province of Orellana in Ecuador and São Paulo state, Brazil. However, despite the identification of similar levels of diversity between the two regions in this study, this is considered to be intraspecific, according to the approaches used, and grouped with the topotype specimen (Accession number: MF381677) from Rio de Janeiro. However, *An. fluminensis* was split into two sympatric lineages (with Ecuadorian specimens and sensu stricto specimens grouped together), which, given their distinct evolutionary histories, may be of varying epidemiological importance. Clearly, *An. fluminensis* has, to date, been understudied and overlooked but, given the species diversity that has been identified in this study from specimens morphologically identified as *An. fluminensis* and the limited sampling effort (only collected from Acre), a more thorough evaluation of lineage diversity and relationships across its considerable South American range is to be encouraged.

Like *An*. *peryassui* and *An. fluminensis*, there are scant genetic data available for *An. costai*. The species appears to be found mainly in forest habitat [78] and is not believed to be an important malaria vector. However, *An. costai* has been frequently misidentified as *An*. *mediopunctatus* [79], which has been found naturally infected with *Plasmodium vivax* in the state of Amazonas [74] and it may yet be recognized as epidemiologically important. This study reveals that *An. costai* exists as a diverse species complex (between 3 and 6 lineages), and several of its lineages have geographical distributions that span the Amazon River basin and beyond. More extensive sampling will be required to determine whether *An. costai* s.s. lineage identified in the clustering-based delimitation occurs in the Amazon River basin, while the ecologies of each species complex member should be explored to determine their potential importance in rural settlements affected by frontier malaria.

*Anopheles punctimacula* ranges from Mexico to Argentina and the Caribbean and has previously been implicated as a malaria vector in Panama [80] and Colombia [81], although this occurred prior to *An*. *malefactor* and *An*. *calderoni* emerging from this taxon [82, 83]. *Anopheles punctimacula* has previously been reported to exist as a species complex, with at least two lineages from Panama supported across multiple genes [36]. The two lineages identified in this study appear to be distantly related to those identified in Loaiza et al. [36], which included the sensu stricto form collected near the type locality in Colón, Panama (and consistently grouped with the Lineage A and B form denoted in that study). One of these lineages exists over quite a considerable range (~ 1000 km; Acre, Brazil—Putumayo, Colombia) and further studies of the phylogeny and ecology of these taxa necesitate employing a range of molecular and morphological markers and more comprehensive sampling in order to more clearly define evolutionary relationships, geographical boundaries, ecological niches and, ultimately, their role in malaria transmission in the region. *Anopheles malefactor*, previously elevated from synonomy with *An. punctimacula* [82], collected from a rural settlement in Acre, was clearly distinct from the *An. malefactor* from the Panama (type locality) and Colombia, and appears to be a new species and sister to a newly delineated *An. fluminensis* G1 lineage.

The close relationships observed among the *Ny. konderi* and *Ny. oswaldoi* complexes indicate that these should be considered a unique species complex named Oswaldoi-Konderi [34]. There are now considered to be five species in this Oswaldoi–Konderi Complex, which include *Ny. oswaldoi* s.s., *Ny. oswaldoi* A, *Ny. oswaldoi* B, *Ny*. *konderi* and *Nyssorhynchus* nr. *konderi* [34, 61]. Although *Ny. konderi* and *Ny*. *oswaldoi* are generally not considered vectors of human malaria, Quiñones et al. [65] found *Ny. oswaldoi* B (although denoted *Ny. oswaldoi* in their study) infected with *P. vivax* in the state of Putumayo, Colombia. The complex may therefore be of epidemiological importance. Although all five species could be effectively delineated in the current study, only four were identified from the collection sites in Acre, Amazonas and Rondônia. *Ny. oswaldoi* s.s. was identified from a site south of Amazonas and (with the inclusion of other data from GenBank) it can also be found Espírito Santo state (type locality; Accession number: JF923721), Paraná, Rondônia and São Paulo. This range covers both the Paraná and Amazon river basins, which are connected by the Parapetí River alluvial fan and may allow fluvial species relatively uninhibited expansion between basins [84]. Whereas *Ny. oswaldoi* A appears to be found through much of the Amazon River basin (Amazonas, Acre, Pará, Rondônia), *Ny. oswaldoi* B was not identified at the collection sites, although it has previously been identified from a site in the northern reaches of the Brazilian Amazon (Santana, Amapá; [34]). A *Ny. oswaldoi* specimen (Field Specimen ID: SP22_9) from Pariquera-Mirim, Pariquera-Açu, São Paulo was included in this study to aid in delineating geographic distribution. However, this specimen was consistently resolved from *Ny. oswaldoi* s.s. and is likely a new species within the Oswaldoi–Konderi Complex. The *Ny. konderi* specimens collected in this study were confined to sites in the state of Acre but this species has also been collected from sites in Amapá (previously denoted *An. konderi* Amapa [60]). Saraiva et al. [34] also identified this species from Amazonas, Pará and Rondônia. With respect to *Ny*. nr. *konderi*, previous studies have found this species in Colombia, Ecuador, Peru [61] and the Brazilian states of Amazonas and Rondônia [34]. In this study, this species was collected from Amazonas but also from a large number of rural settlements in Acre.

Recent studies have confirmed *Ny. benarrochi* as a species complex, with the identification of *Ny. benarrochi* B in Colombia [85] and Peru [86]. Although *Ny. benarrochi* is predominantly zoophilic [87] and therefore not believed to be an important malaria vector, highly anthropophilic behaviour does occur in some areas and it may be play a role in malaria transmission in the Peruvian Amazon [85, 88]. This study confirms the existence of *Ny. benarrochi* B, using data from Conn et al. [86], in rural settlements in the state of Acre. It also identifies at least one other *Ny*. *benarrochi* lineage from Acre and Rondônia (and perhaps representing two). Unfortunately, this lineage cannot be confirmed as *Ny. benarrochi* s.s. as material from the type locality in the municipality of La Ceiba, Trujillo State, Venezuela is unavailable. It is, therefore, essential that collections be obtained from this type locality in order to undertake a meaningful systematic review of the *Ny*. *benarrochi* complex and determine whether species diversity within this complex is associated with variation in anthropophilia and vector competence.

*Nyssorhynchus triannulatus* is found throughout Central and South America, and has been incriminated in the transmission of human malaria in the states of Amapa [67], Amazonas [74] and Pará [68], Brazil. Recent studies have shown that *Ny. triannulatus* forms a species complex with two other recently identified species; *Ny. halophylus* [89] and *Ny. triannulatus* C [90]. *Nyssorhynchus triannulatus* sensu lato has been found to form paraphyletic (at a combined ITS2, *white* and *COI* gene tree; [91]) and monophyletic sister [using RAPD markers; 92] relationships with an *Ny*. *halophylus* and *Ny. triannulatus* C clade. Within *Ny*. *triannulatus* s.l. two further clades have been recovered with the *COI* gene, one from Central America and northern Colombia and the other primarily from the Amazon river basin [91], and several additional biologically meaningful subclades from within the Amazonian clade were also proposed, based on multiple gene (*COI*, *white* and ITS2) and haplotype network analysis. The haplotypes included in this study come from the Amazonian states of Acre and Amapá, as well as from the states of Minas Gerais to the east, Espírito Santo on the Atlantic coast, São Paulo in the south east of Brazil, and neighbouring Colombia. Despite a considerable geographic distribution and habitat range [91, 92], results from the current analysis show that, although the *Ny. triannulatus* s.l. clade is extremely diverse, relationships among its *COI* haplotypes cannot be meaningfully partitioned into separate species i.e., all branch structure and pairwise distances within *Ny. triannulatus* are considered intraspecific. It appears that relationships may be better explained by complex population histories [92], where elevated levels of genetic diversity have been maintained perhaps by historical fragmentation, secondary contact and gene-flow followed by more recent divergence due to geographical isolation. Further phylogenetic and species delimitation analyses at multiple loci may reveal more compelling support for species designation within *Ny. triannulatus*, as phylogenetic analysis of *COI* frequently fails to resolve well supported species of Anophelinae mosquitoes, e.g., *Ny. strodei* and *Ny. albertoi*. Conflict among gene trees due to incomplete lineage sorting and horizontal gene transfer (gene-flow) is pervasive in very closely related species, making molecular identification of such species difficult. High density codominant markers such as single nucleotide polymorphisms (SNPs) offer some opportunities to overcome such phylogenetic challenges [93–96] and an exploration of the hierarchy of structure within *Ny. triannulatus* (and *Ny. braziliensis*) may be better achieved using such markers in combination with population genetic approaches [97].

Several of the morphospecies collected in this study are highly diverse complexes, the diversity of which is supported by a range of ecological, morphological and genetic data. However, the use of species delimitation approaches with the *COI* gene failed to detect several of these species boundaries. Currently, 10 species have been identified within the *Ny. albitarsis* complex, five of which (*Ny. albitarsis*, *Ny. albitarsis* H, *An. deaneorum*, *Ny. marajoara* and *Ny. oryzalimnetes*) were collected in this study. A range of morphological and molecular data has been employed to resolve and support species in this complex [98–100]. Nevertheless, the close relationships observed among *Ny. albitarsis* H, *Ny. deaneorum* and *Ny. marajoara* at the *COI* gene in the present study were consistent with patterns of intraspecific variation when employing cluster- and tree-based species delimitation. It appears that species delimitation of this complex at the *COI* gene will fail to detect important species boundaries supported by other data and further exploration and discovery of species diversity in the Albitarsis Complex will require a multilocus approach, possibly using loci such as ITS2 and the *white* gene, which have been used to resolve many of the species within the complex [101, 102].

Similar issues were encountered when dealing with the Nuneztovari Complex and Strodei Subgroup. The former is comprised of *Ny*. *dunhami*, *Ny. goeldii*, *Ny*. *nuneztovari* [103], supported by a range of morphological and molecular data [104–106]. Its geographical distribution runs from the Isthmus of Panama to northern South America and *Ny. nuneztovari* sensu lato is considered one of the most important malaria vectors in the region [26]. A recent study by Scarpassa et al. [40] found *Ny*. *nuneztovari * s.l. haplotypes were variously identified as *Ny. dunhami*, *Ny*. *goeldii*, *Ny. nuneztovari* and an additional unknown clade. They found that population genetic and phylogenetic analysis were in some agreement, although lineage delineation and relationships among *COI* trees that differed in sampling effort were to some degree incongruent. Although the current study combined the sequences from Scarpassa et al. [40] with those from specimens collected from the Brazilian Amazonian states of Acre, Amapá, Amazonas, Pará, Rondônia, it failed to delineate any species within the *Nyssorhynchus nuneztovari* complex. The Strodei Subgroup is made up of *Ny. albertoi*, *Ny. strodei* [107], *Ny. striatus* [108], *Ny. rondoni* and finally *Ny. arthuri*, which is comprised of four species A - D [35]. Given the systematic complexity of this group and the recent emergence of many of its species, the importance of these species in malaria transmission is unknown. However, *Ny*. *strodei* has previously been found naturally infected with *P*. *vivax* in Ariquemes, Rondônia [73], although this case may refer to *Ny. arthuri* C [35]. *Nyssorhynchus albertoi* and *Ny. strodei*, despite having considerable support as separate species within the Strodei Subgroup [107], are known to be unresolved with the *COI* gene [35] and, unsurprisingly, species delimitation in the current study using this same gene failed to identify the respective species. The other members of the subgroup included in the study were resolved and clearly delineated. As in Bourke et al. [35], *Ny. arthuri* A was not detected from the Amazon region and *Ny. arthuri* C has so far only been identified from the state of Rondônia. Further analysis of this species range and habitat is required before its epidemiological importance in rural settlements can be determined.

The work presented here demonstrates a clear advantage to employing species delimitation approaches when the objective is to explore species diversity and discover new species in Anophelinae where cryptic species boundaries are common. Due to the relative speed at which *COI* can be sequenced and analysed, the study demonstrates the power of single-locus species delimitation approaches to establish a baseline of species diversity in Anophelinae in remote and unexplored regions of the Amazon River basin. However, it must be clearly stated that single-locus data alone should only be used to provide a preliminary description of species boundaries (possible gene tree-species tree discordance) and the new species delimited in this study remain putative. Rather, it allows for the establishment of viable species hypotheses, to be tested against a range on independent data sources that may be, for example, morphological, molecular and/or ecological in nature. In particular, there have been few or no classical studies of Anophelinae taxonomy conducted in the Amazon River basin in recent years, and further studies of species diversity in Anophelinae are, therefore, encouraged to better explore morphological variation among these species. Future work on species exploration and discovery in the Amazon River basin should also employ a multilocus delimitation approach [43, 109] to better enable the resolution of all recognized species within important complexes, such as Albitarsis and Nuneztovari. In addition, the study demonstrates that exceptional diversity detected in morphospecies can be consistent with a model of intraspecific diversity and is suggestive of a complex evolutionary history that may be better explored using high-density markers and population genetic approaches.

This study has been successful in revealing a large number of unknown Anophelinae species that are likely to be new to science and occur in areas with endemic malaria transmission. One of the great challenges for malaria control in the Amazon River basin is the transmission that occurs outside of rural dwellings and the detection of new species that belong to groups containing important vectors will, therefore, have an important impact on the development of effective vector management and control strategies in the region. The World Health Organization recommends that vector management and control interventions should take account of potential impacts on the environment and biodiversity and should be focused on avoiding unintended impacts on non-vector species. The findings from this study will assist in refining such strategies, help build capacity in public health entomology and provide an important contribution to effective malaria control in the region.

## Conclusion

Anophelinae mosquito diversity continues to be underestimated in poorly sampled tropical rain forest areas where frontier malaria is a major public health concern. Delimitation approaches used to explore Anophelinae mosquito diversity in these areas reveal a large number of unknown species that are likely to be new to science. These findings will help shape future studies of vector incrimination and transmission dynamics and support efforts to develop more effective vector control and transmission reduction strategies in settler communities in the Amazon River basin.

## Additional files

**Additional file 1.** Sample information of collection specimens and reference sequences.

**Additional file 2.** *COI* sequence data for all collection specimens and references used in this study.

**Additional file 3.** Output from mPTP analysis of collection specimens and reference sequences.

**Additional file 4.** “Strict” partition from ABGD analysis of collection specimens and reference sequences.

**Additional file 5.** “Relaxed” partition from ABGD analysis of collection specimens and reference sequences.

## References

[1] Instituto Nacional de Pesquisas Espaciais. Annual deforestation rate in the Brazilian Legal Amazon. 2018. http://www.obt.inpe.br/prodes/dashboard/prodes-rates.html. Accessed 3 Mar 2018.

[2] Garcia B (2011). The characteristics of the Amazon Region. The Amazon from an international law perspective.

[3] Barlow J, Lennox GD, Ferreira J, Berenguer E, Lees AC, Mac Nally R (2016). Anthropogenic disturbance in tropical forests can double biodiversity loss from deforestation. Nature.

[4] Menezes JA, Confalonieri U, Madureira AP, de Brito Duval I, Santos RBD, Margonari C (2018). Mapping human vulnerability to climate change in the Brazilian Amazon: the construction of a municipal vulnerability index. PLoS ONE.

[5] Walsh JF, Molyneux DH, Birley MH (1993). Deforestation: effects on vector-borne disease. Parasitology.

[6] Camargo LM, Ferreira MU, Krieger H, De Camargo EP, Da Silva LP (1994). Unstable hypoendemic malaria in Rondonia (western Amazon region, Brazil): epidemic outbreaks and work-associated incidence in an agro-industrial rural settlement. Am J Trop Med Hyg.

[7] Vasconcelos PF, Rodrigues SG, Degallier N, Moraes MA, da Rosa JF, da Rosa ES (1997). An epidemic of sylvatic yellow fever in the southeast region of Maranhao State, Brazil, 1993–1994: epidemiologic and entomologic findings. Am J Trop Med Hyg.

[8] Nava A, Shimabukuro JS, Chmura AA, Luz SLB (2017). The impact of global environmental changes on infectious disease emergence with a focus on risks for Brazil. ILAR J.

[9] Lima JMT, Vittor A, Rifai S, Valle D (2017). Does deforestation promote or inhibit malaria transmission in the Amazon? A systematic literature review and critical appraisal of current evidence. Philos Trans R Soc Lond B Biol Sci.

[10] Ferreira MU, Castro MC (2016). Challenges for malaria elimination in Brazil. Malar J.

[11] Hahn MB, Gangnon RE, Barcellos C, Asner GP, Patz JA (2014). Influence of deforestation, logging, and fire on malaria in the Brazilian Amazon. PLoS ONE.

[12] Marques AC (1987). Human migration and the spread of malaria in Brazil. Parasitol Today.

[13] WHO (2017). World malaria report.

[14] Sawyer DR (1988). Frontier malaria in the Amazon region of Brazil: types of malaria situations and some implications for control.

[15] Castro MC, Monte-Mór RL, Sawyer DO, Singer BH (2006). Malaria risk on the Amazon frontier. Proc Natl Acad Sci USA.

[16] Hiwat H, Bretas G (2011). Ecology of *Anopheles darlingi* root with respect to vector importance: a review. Parasites Vectors.

[17] Castro MC, Singer BH, Selendy JMH (2011). Malaria in the Brazilian Amazon. Water and sanitation related diseases and the environment: challenges, interventions, and preventive measures.

[18] Cheng Q, Cunningham J, Gatton ML (2015). Systematic review of sub-microscopic *P. vivax* infections: prevalence and determining factors. PLoS Negl Trop Dis.

[19] White NJ, Imwong M (2012). Relapse. Adv Parasitol.

[20] Ashley EA, Recht J, White NJ (2014). Primaquine: the risks and the benefits. Malar J.

[21] Watson J, Taylor WRJ, Bancone G, Chu CS, Jittamala P, White NJ (2018). Implications of current therapeutic restrictions for primaquine and tafenoquine in the radical cure of vivax malaria. PLoS Negl Trop Dis.

[22] Sampaio VS, Siqueira AM, Alecrim MDGC, Mourão MPG, Marchesini PB, Albuquerque BC (2015). Malaria in the State of Amazonas: a typical Brazilian tropical disease influenced by waves of economic development. Rev Soc Bras Med Trop.

[23] Conn JE, Quiñones ML, Póvoa MM, Manguin S (2013). Phylogeography, vectors and transmission in Latin America. Anopheles mosquitoes: new insights into malaria vectors.

[24] Rios-Velásquez CM, Martins-Campos KM, Simões RC, Izzo T, dos Santos EV, Pessoa FAC (2013). Experimental *Plasmodium vivax* infection of key Anopheles species from the Brazilian Amazon. Malar J.

[25] da Silva-Vasconcelos A, Kató MYN, Mourão EN, de Souza RTL, Lacerda RNDL, Sibajev A (2002). Biting indices, host-seeking activity and natural infection rates of anopheline species in Boa Vista, Roraima, Brazil from 1996 to 1998. Mem Inst Oswaldo Cruz.

[26] Sinka ME, Rubio-Palis Y, Manguin S, Patil AP, Temperley WH, Gething PW (2010). The dominant Anopheles vectors of human malaria in the Americas: occurrence data, distribution maps and bionomic précis. Parasites Vectors.

[27] Foster PG, de Oliveira TMP, Bergo ES, Conn JE, Sant’Ana DC, Nagaki SS (2017). Phylogeny of Anophelinae using mitochondrial protein coding genes. R Soc Open Sci.

[28] Vittor AY, Gilman RH, Tielsch J, Glass G, Shields T, Lozano WS (2006). The effect of deforestation on the human-biting rate of *Anopheles darlingi*, the primary vector of Falciparum malaria in the Peruvian Amazon. Am J Trop Med Hyg.

[29] Vittor AY, Pan W, Gilman RH, Tielsch J, Glass G, Shields T (2009). Linking deforestation to malaria in the Amazon: characterization of the breeding habitat of the principal malaria vector, *Anopheles darlingi*. Am J Trop Med Hyg.

[30] Reis IC, Codeço CT, Câmara DCP, Carvajal JJ, Pereira GR, Keppeler EC (2018). Diversity of *Anopheles* spp. (Diptera: Culicidae) in an Amazonian urban area. Neotrop Entomol.

[31] Gómez GF, Bickersmith SA, González R, Conn JE, Correa MM (2015). Molecular taxonomy provides new insights into Anopheles species of the neotropical Arribalzagia series. PLoS ONE.

[32] Tadei WP, Thatcher BD, Santos JM, Scarpassa VM, Rodrigues IB, Rafael MS (1998). Ecologic observations on anopheline vectors of malaria in the Brazilian Amazon. Am J Trop Med Hyg.

[33] Rosa-Freitas MG, Lourenço-de-Oliveira R, de Carvalho-Pinto CJ, Flores-Mendoza C, Silva-do-Nascimento TF (1998). Anopheline species complexes in Brazil. Current knowledge of those related to malaria transmission. Mem Inst Oswaldo Cruz.

[34] Saraiva JF, Souto RNP, Scarpassa VM (2018). Molecular taxonomy and evolutionary relationships in the Oswaldoi–Konderi complex (Anophelinae: Anopheles: Nyssorhynchus) from the Brazilian Amazon region. PLoS ONE.

[35] Bourke BP, Oliveira TP, Suesdek L, Bergo ES, Sallum MAM (2013). A multi-locus approach to barcoding in the *Anopheles strodei* subgroup (Diptera: Culicidae). Parasit Vectors.

[36] Loaiza JR, Scott ME, Bermingham E, Sanjur OI, Rovira JR, Dutari LC (2013). Novel genetic diversity within *Anopheles punctimacula* s.l.: phylogenetic discrepancy between the Barcode cytochrome c oxidase I (COI) gene and the rDNA second internal transcribed spacer (ITS2). Acta Trop.

[37] Barbosa LMC, Souto RNP, Dos Anjos Ferreira RM, Scarpassa VM (2016). Behavioral patterns, parity rate and natural infection analysis in anopheline species involved in the transmission of malaria in the northeastern Brazilian Amazon region. Acta Trop.

[38] Barros FSM, Honório NA, Arruda ME (2010). Mosquito anthropophily: implications on malaria transmission in the Northern Brazilian Amazon. Neotrop Entomol.

[39] Vogler AP, Monaghan MT (2007). Recent advances in DNA taxonomy. J Zoolog Syst Evol Res.

[40] Scarpassa VM, Cunha-Machado AS, Saraiva JF (2016). Evidence of new species for malaria vector *Anopheles nuneztovari* sensu lato in the Brazilian Amazon region. Malar J.

[41] González R, Carrejo N, Wilkerson RC, Alarcon J, Alarcon-Ormasa J, Ruiz F (2010). Confirmation of *Anopheles* (Anopheles) *calderoni* Wilkerson, 1991 (Diptera: Culicidae) in Colombia and Ecuador through molecular and morphological correlation with topotypic material. Mem Inst Oswaldo Cruz.

[42] Collins RA, Cruickshank RH (2013). The seven deadly sins of DNA barcoding. Mol Ecol Resour.

[43] Leavitt SD, Moreau CS, Thorsten Lumbsch H (2015). The dynamic discipline of species delimitation: progress toward effectively recognizing species boundaries in natural populations. Recent advances in lichenology.

[44] Puillandre N, Lambert A, Brouillet S, Achaz G (2012). ABGD, Automatic Barcode Gap Discovery for primary species delimitation. Mol Ecol.

[45] Kapli P, Lutteropp S, Zhang J, Kobert K, Pavlidis P, Stamatakis A (2017). Multi-rate Poisson tree processes for single-locus species delimitation under maximum likelihood and Markov chain Monte Carlo. Bioinformatics.

[46] Folmer O, Black M, Hoeh W, Lutz R, Vrijenhoek R (1994). DNA primers for amplification of mitochondrial cytochrome c oxidase subunit I from diverse metazoan invertebrates. Mol Mar Biol Biotechnol.

[47] Zapata S, Mejía L, Le Pont F, León R, Pesson B, Ravel C (2012). A study of a population of *Nyssomyia trapidoi* (Diptera: Psychodidae) caught on the Pacific coast of Ecuador. Parasites Vectors.

[48] Edgar RC (2004). MUSCLE: multiple sequence alignment with high accuracy and high throughput. Nucleic Acids Res.

[49] Gouy M, Guindon S, Gascuel O (2010). SeaView version 4: a multiplatform graphical user interface for sequence alignment and phylogenetic tree building. Mol Biol Evol.

[50] Abascal F, Zardoya R, Telford MJ (2010). TranslatorX: multiple alignment of nucleotide sequences guided by amino acid translations. Nucleic Acids Res.

[51] Darriba D, Taboada GL, Doallo R, Posada D (2012). jModelTest 2: more models, new heuristics and parallel computing. Nat Methods.

[52] Ronquist F, Huelsenbeck JP (2003). MrBayes 3: Bayesian phylogenetic inference under mixed models. Bioinformatics.

[53] Miller MA, Pfeiffer W, Schwartz T. Creating the CIPRES Science Gateway for inference of large phylogenetic trees. In: 2010 gateway computing environments workshop (GCE). 2010. 10.1109/gce.2010.5676129.

[54] Sukumaran J, Holder MT. SumTrees: phylogenetic tree summarization. 2015. https://github.com/jeetsukumaran/DendroPy. Accessed 10 Feb 2018.

[55] Sukumaran J, Holder MT (2010). DendroPy: a Python library for phylogenetic computing. Bioinformatics.

[56] Kimura M (1980). A simple method for estimating evolutionary rates of base substitutions through comparative studies of nucleotide sequences. J Mol Evol.

[57] Paradis E, Claude J, Strimmer K (2004). APE: analyses of phylogenetics and evolution in R language. Bioinformatics.

[58] Stamatakis A (2014). RAxML version 8: a tool for phylogenetic analysis and post-analysis of large phylogenies. Bioinformatics.

[59] Linton Y-M, Pecor JE, Porter CH, Mitchell LB, Garzón-Moreno A, Foley DH (2013). Mosquitoes of eastern Amazonian Ecuador: biodiversity, bionomics and barcodes. Mem Inst Oswaldo Cruz.

[60] Motoki MT, Bourke BP, Bergo ES, Da Silva AM, Sallum MAM (2011). Systematic notes of *Anopheles konderi* and its first record in Paraná State, Brazil. J Am Mosq Control Assoc.

[61] Ruiz-Lopez F, Wilkerson RC, Ponsonby DJ, Herrera M, Sallum MAM, Velez ID (2013). Systematics of the oswaldoi complex (Anopheles, Nyssorhynchus) in South America. Parasites Vectors.

[62] Chaves LSM, Conn JE, López RVM, Sallum MAM (2018). Abundance of impacted forest patches less than 5 km is a key driver of the incidence of malaria in Amazonian Brazil. Sci Rep.

[63] Prussing C, Moreno M, Saavedra MP, Bickersmith SA, Gamboa D, Alava F (2018). Decreasing proportion of *Anopheles darlingi* biting outdoors between long-lasting insecticidal net distributions in peri-Iquitos, Amazonian Peru. Malar J.

[64] Conn JE, Ribolla PE, Adelman Z (2016). Ecology of *Anopheles darlingi*, the primary malaria vector in the Americas and current nongenetic methods of vector control. Genetic control of malaria and dengue.

[65] Quiñones ML, Ruiz F, Calle DA, Harbach RE, Erazo HF, Linton Y-M (2006). Incrimination of *Anopheles* (*Nyssorhynchus*) *rangeli* and *An.* (*Nys.*) *oswaldoi* as natural vectors of *Plasmodium vivax* in Southern Colombia. Mem Inst Oswaldo Cruz.

[66] Wermelinger ED, Benigno CV, Machado RNM, Nascimento TFS, Ferreira AP, Meira AM (2010). Occurrence of Anopheles (Nyssorhynchus) rangeli (Gabaldon et al.) and Anopheles (Nyssorhynchus) evansae (Brethes) (Diptera: Culicidae) in an Eutrophized Dam. Neotrop Entomol.

[67] Galardo AKR, Arruda M, D’Almeida Couto AAR, Wirtz R, Lounibos LP, Zimmerman RH (2007). Malaria vector incrimination in three rural riverine villages in the Brazilian Amazon. Am J Trop Med Hyg.

[68] de Arruda M, Carvalho MB, Nussenzweig RS, Maracic M, Ferreira AW, Cochrane AH (1986). Potential vectors of malaria and their different susceptibility to *Plasmodium falciparum* and *Plasmodium vivax* in northern Brazil identified by immunoassay. Am J Trop Med Hyg.

[69] Dusfour I, Issaly J, Carinci R, Gaborit P, Girod R (2012). Incrimination of *Anopheles* (*Anopheles*) *intermedius* Peryassú, *An.* (*Nyssorhynchus*) *nuneztovari* Gabaldón, *An.* (*Nys.*) *oswaldoi* Peryassú as natural vectors of *Plasmodium falciparum* in French Guiana. Mem Inst Oswaldo Cruz.

[70] Póvoa MM, Wirtz RA, Lacerda R, Miles MA, Warhurst D (2001). Malaria vectors in the municipality of Serra do Navio, State of Amapá, Amazon Region, Brazil. Mem Inst Oswaldo Cruz.

[71] Morais SA, Urbinatti PR, Sallum MAM, Kuniy AA, Moresco GG, Fernandes A (2012). Brazilian mosquito (Diptera: Culicidae) fauna: I. Anopheles species from Porto Velho, Rondônia state, western Amazon, Brazil. Rev Inst Med Trop Sao Paulo.

[72] Póvoa MM, de Souza RTL, Lacerda RNDL, Rosa ES, Galiza D, de Souza JR (2006). The importance of *Anopheles albitarsis* E and *An. darlingi* in human malaria transmission in Boa Vista, state of Roraima, Brazil. Mem Inst Oswaldo Cruz.

[73] de Oliveira-Ferreira J, Daniel-Ribeiro CT, Lourenço-De-Oliveira R, Teva A, Deane LM (1990). Natural malaria infections in Anophelines in Rondonia State, Brazilian Amazon. Am J Trop Med Hyg.

[74] Tadei WP, Dutary Thatcher B (2000). Malaria vectors in the Brazilian Amazon: anopheles of the subgenus Nyssorhynchus. Rev Inst Med Trop Sao Paulo.

[75] Cerqueira NL (1961). Distribuição geográfica dos mosquitos da Amazônia. Rev Bras Entomol.

[76] Hayes J, Calderon G, Falcon R, Zambrano V (1987). Newly incriminated anopheline vectors of human malaria parasites in Junin Department, Peru. J Am Mosq Control Assoc.

[77] Neves A, Urbinatti PR, dos Santos Malafronte R, Fernandes A, da Silva Paganini W, Natal D (2013). Malaria outside the Amazon region: natural Plasmodium infection in anophelines collected near an indigenous village in the Vale do Rio Branco, Itanhaém, SP, Brazil. Acta Trop.

[78] Da Silva KS, Pinto IDS, Leite GR, Das Virgens TM, Dos Santos CB, Falqueto A (2013). Ecology of anopheline mosquitoes (Diptera: Culicidae) in the Central Atlantic Forest Biodiversity Corridor, southeastern Brazil. J Med Entomol.

[79] Sallum MA, Wilkerson RC, Forattini OP (1999). Taxonomic study of species formerly identified as *Anopheles mediopunctatus* and resurrection of *An. costai* (Diptera: Culicidae). J Med Entomol.

[80] Simmons JS (1936). *Anopheles* (*Anopheles*) *punctimacula* naturally infected with malaria Plasmodia. Am J Trop Med Hyg.

[81] Rey H, Huffaker CB, Soto H (1945). Anopheles punctimacula D. & K. as the vector of malaria in Medellín, Colombia, South America 1,2. Am J Trop Med Hyg.

[82] Wilkerson RC (1990). Redescriptions of *Anopheles punctimacula* and *An. malefactor* (Diptera: Culicidae). J Med Entomol.

[83] Wilkerson RC (1991). *Anopheles* (*Anopheles*) *calderoni* sp., a malaria vector of the Arribalzagia series from Peru. Mosq Syst.

[84] Iriondo MH, Paira AR, Iriondo MH, Paggi JC, Parma MJ (2007). Physical geography of the basin. The Middle Parana River limnology of a subtropical wetland.

[85] Ruiz F, Quiñones ML, Erazo HF, Calle DA, Alzate JF, Linton Y-M (2005). Molecular differentiation of *Anopheles* (*Nyssorhynchus*) *benarrochi* and *An.* (*N.*) *oswaldoi* from southern Colombia. Mem Inst Oswaldo Cruz.

[86] Conn JE, Moreno M, Saavedra M, Bickersmith SA, Knoll E, Fernandez R (2013). Molecular taxonomy of *Anopheles* (*Nyssorhynchus*) *benarrochi* (Diptera: Culicidae) and malaria epidemiology in southern Amazonian Peru. Am J Trop Med Hyg.

[87] Faran ME (1980). Mosquito studies (Diptera, Culicidae) XXXIV. A revision of the Albimanus section of the subgenus Nyssorhynchus of Anopheles. Contrib Am Entomol Inst.

[88] Flores-Mendoza C, Fernández R, Escobedo-Vargas KS, Vela-Perez Q, Schoeler GB (2004). Natural Plasmodium infections in *Anopheles darlingi* and *Anopheles benarrochi* (Diptera: Culicidae) from eastern Peru. J Med Entomol.

[89] Nascimento TFSD, Lourenco-de-Oliveira R (2002). Anopheles halophylus, a new species of the subgenus Nyssorhynchus (Diptera: Culicidae) from Brazil. Mem Inst Oswaldo Cruz.

[90] Silva-Do-Nascimento TF, Wilkerson RC, Lourenço-De-oliveira R, Monteiro FA (2006). Molecular confirmation of the specific status of *Anopheles halophylus* (Diptera: Culicidae) and evidence of a new cryptic species within *An. triannulatus* in central Brazil. J Med Entomol.

[91] Moreno M, Bickersmith S, Harlow W, Hildebrandt J, McKeon SN, Silva-do-Nascimento TF (2013). Phylogeography of the neotropical *Anopheles triannulatus* complex (Diptera: Culicidae) supports deep structure and complex patterns. Parasites Vectors.

[92] McKeon SN, Moreno M, Sallum MA, Povoa MM, Conn JE (2013). Distinct population structure for co-occurring *Anopheles goeldii* and *Anopheles triannulatus* in Amazonian Brazil. Mem Inst Oswaldo Cruz.

[93] Eaton DAR, Ree RH (2013). Inferring phylogeny and introgression using RADseq data: an example from flowering plants (Pedicularis: Orobanchaceae). Syst Biol.

[94] Wagner CE, Keller I, Wittwer S, Selz OM, Mwaiko S, Greuter L (2013). Genome-wide RAD sequence data provide unprecedented resolution of species boundaries and relationships in the Lake Victoria cichlid adaptive radiation. Mol Ecol.

[95] Cruaud A, Gautier M, Galan M, Foucaud J, Sauné L, Genson G (2014). Empirical assessment of RAD sequencing for interspecific phylogeny. Mol Biol Evol.

[96] Andrews KR, Good JM, Miller MR, Luikart G, Hohenlohe PA (2016). Harnessing the power of RADseq for ecological and evolutionary genomics. Nat Rev Genet.

[97] Baumsteiger J, Moyle PB, Aguilar A, O’Rourke SM, Miller MR (2017). Genomics clarifies taxonomic boundaries in a difficult species complex. PLoS ONE.

[98] Ruiz-Lopez F, Wilkerson RC, Conn JE, McKeon SN, Levin DM, Quiñones ML (2012). DNA barcoding reveals both known and novel taxa in the Albitarsis group (Anopheles: Nyssorhynchus) of Neotropical malaria vectors. Parasites Vectors.

[99] Bourke BP, Foster PG, Bergo ES, Calado DC, Sallum MAM (2010). Phylogenetic relationships among species of Anopheles (Nyssorhynchus) (Diptera, Culicidae) based on nuclear and mitochondrial gene sequences. Acta Trop.

[100] Motoki MT, Wilkerson RC (2009). Sallum MAM. The *Anopheles albitarsis* complex with the recognition of *Anopheles oryzalimnetes* Wilkerson and Motoki, n. sp. and *Anopheles janconnae* Wilkerson and Sallum, n. sp. (Diptera: Culicidae). Mem Inst Oswaldo Cruz.

[101] Krzywinski J, Li C, Morris M, Conn JE, Lima JB, Povoa MM (2011). Analysis of the evolutionary forces shaping mitochondrial genomes of a Neotropical malaria vector complex. Mol Phylogenet Evol.

[102] Brochero HHL, Li C, Wilkerson RC (2007). A newly recognized species in the *Anopheles (Nyssorhynchus) albitarsis* complex (Diptera: Culicidae) from Puerto Carreno, Colombia. Am J Trop Med Hyg..

[103] Foster PG, Bergo ES, Bourke BP, Oliveira TMP, Nagaki SS, Sant’Ana DC (2013). Phylogenetic analysis and DNA-based species confirmation in Anopheles (Nyssorhynchus). PLoS ONE.

[104] Peyton EL (1993). *Anopheles* (*Nyssorhynchus*) *dunhami*, resurrected from synonymy with *Anopheles nuneztovari* and validated as a senior synonym of *Anopheles trinkae* (Diptera: Culicidae). Mosq Syst.

[105] Calado DC, Foster PG, Bergo ES, dos Santos CLS, Galardo AKR, Sallum MAM (2008). Resurrection of *Anopheles goeldii* from synonymy with *Anopheles nuneztovari* (Diptera, Culicidae) and a new record for *Anopheles dunhami* in the Brazilian Amazon. Mem Inst Oswaldo Cruz.

[106] Sant’Ana DC, Bergo ES, Sallum MAM (2015). *Anopheles goeldii* Rozeboom & Gabaldón (Diptera, Culicidae): a species of the Nuneztovari complex of Anopheles Meigen. Rev Bras Entomol.

[107] Sallum MAM, Foster PG, Dos Santos CLS, Flores DC, Motoki MT, Bergo ES (2010). Resurrection of two species from synonymy of *Anopheles* (*Nyssorhynchus*) *strodei* Root, and characterization of a distinct morphological form from the Strodei complex (Diptera: Culicidae). J Med Entomol.

[108] Sant’Ana DC, Sallum MAM (2017). *Anopheles* (*Nyssorhynchus*) *striatus*, a new species of the Strodei Subgroup (Diptera, Culicidae). Rev Bras Entomol.

[109] Fujita MK, Leaché AD, Burbrink FT, McGuire JA, Moritz C (2012). Coalescent-based species delimitation in an integrative taxonomy. Trends Ecol Evol.

